# P-1369. Infectious Disease Provider Perspectives on Shorter Tuberculosis Treatment Regimens

**DOI:** 10.1093/ofid/ofaf695.1556

**Published:** 2026-01-11

**Authors:** Aliya Moreira, Dana Hassneiah, Susan E Beekmann, Philip M Polgreen, Maunank Shah, Ruvandhi Nathavitharana

**Affiliations:** Beth Israel Deaconess Medical Center, Brookline, MA; Beth Israel Deaconess Medical Center, Brookline, MA; University of Iowa, IOWA CITY, Iowa; University of Iowa Carver College of Medicine, Iowa City, IA; Johns Hopkins, Baltimore, MD; Beth Israel Deaconess Medical Center, Brookline, MA

## Abstract

**Background:**

Since 2020, CDC guidance has preferentially recommended shorter regimens for tuberculosis (TB) infection and since 2022, recommendations include 6-month regimens for drug-resistant (DR-TB) disease containing bedaquiline, linezolid, and pretomanid (BPaL) and a 4-month regimen for drug-susceptible (DS-TB) disease containing high-dose rifapentine, isoniazid, moxifloxacin, and pyrazinamide (HPMZ). Yet guideline implementation often lags. This survey aimed to characterize TB treatment practices of North American infectious disease (ID) physicians and barriers to the utilization of shorter regimens.

**Table 1:**
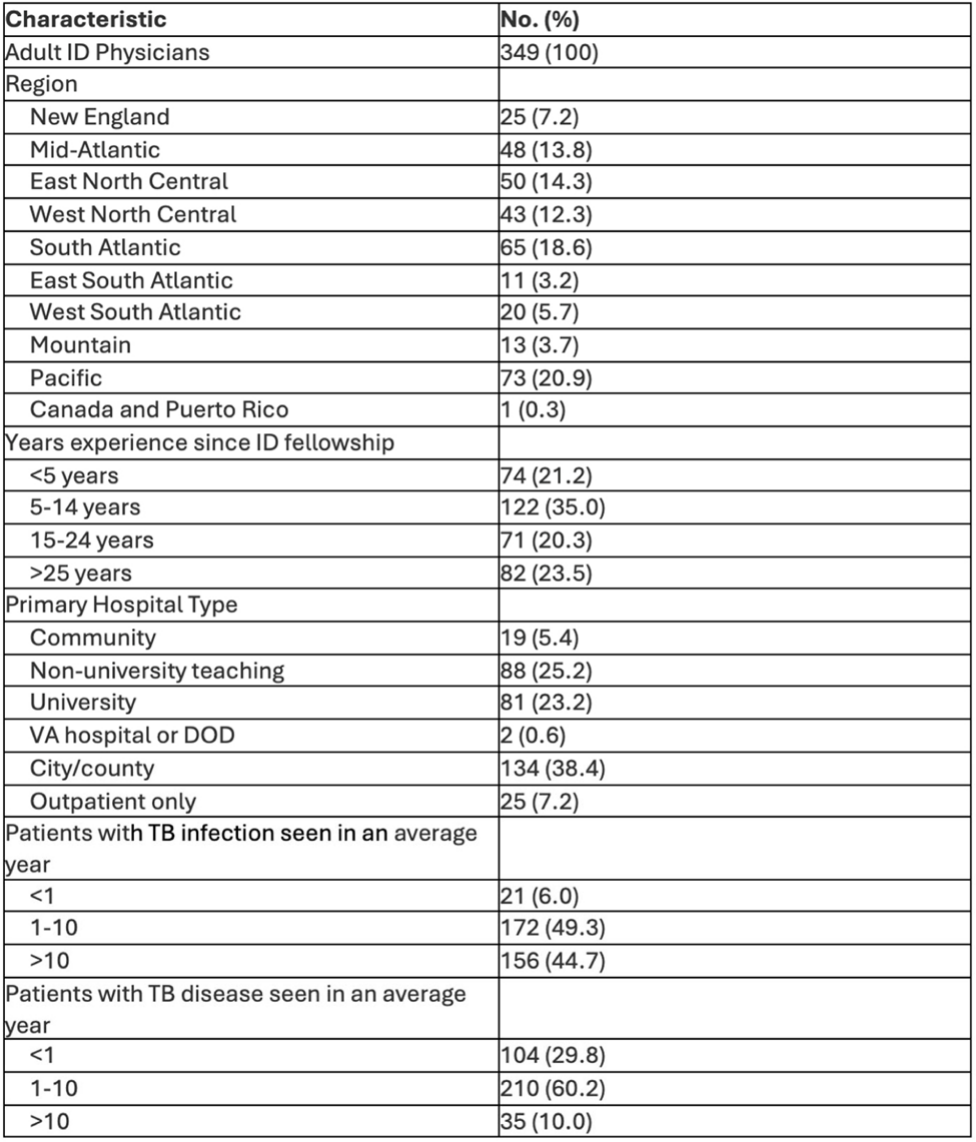
Demographics

**Figure 1:**
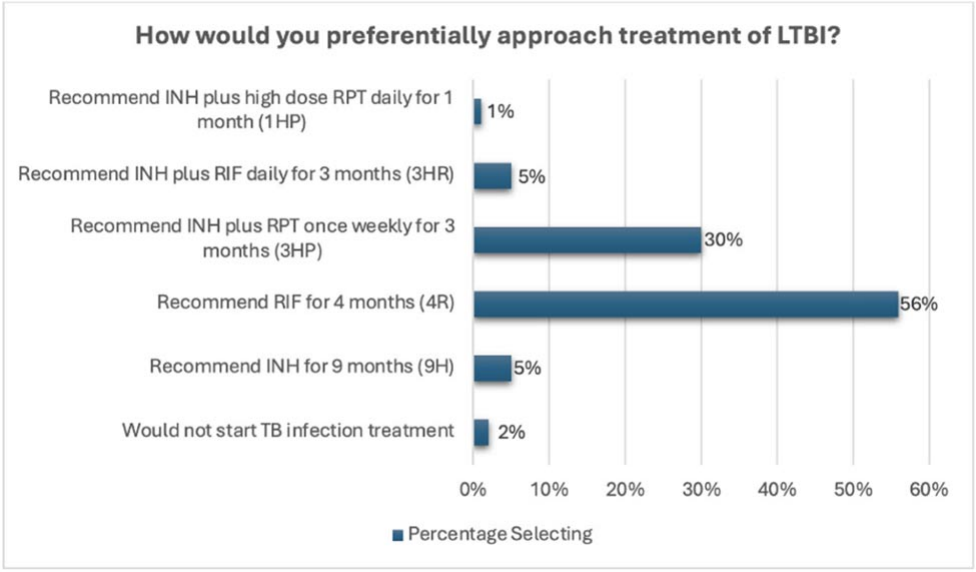
Participant selected preferential approaches to managing LTBI

**Methods:**

A survey about TB treatment practices was distributed to 1501 North American adult ID physician members of the IDSA Emerging Infections Network.

The survey used hypothetical case scenarios to elicit treatment preferences for TB infection, DS-TB disease, and DR-TB disease. Descriptive data analyses were performed using Excel. Open comment question data were qualitatively analyzed.

**Figure 2:**
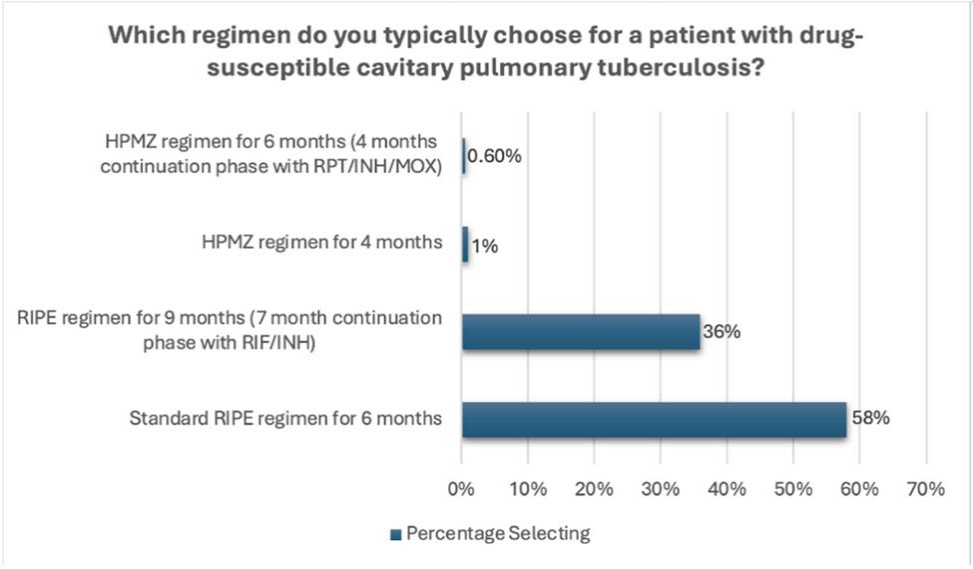
Participant selected preferential approaches to managing drug-susceptible TB

**Figure 3:**
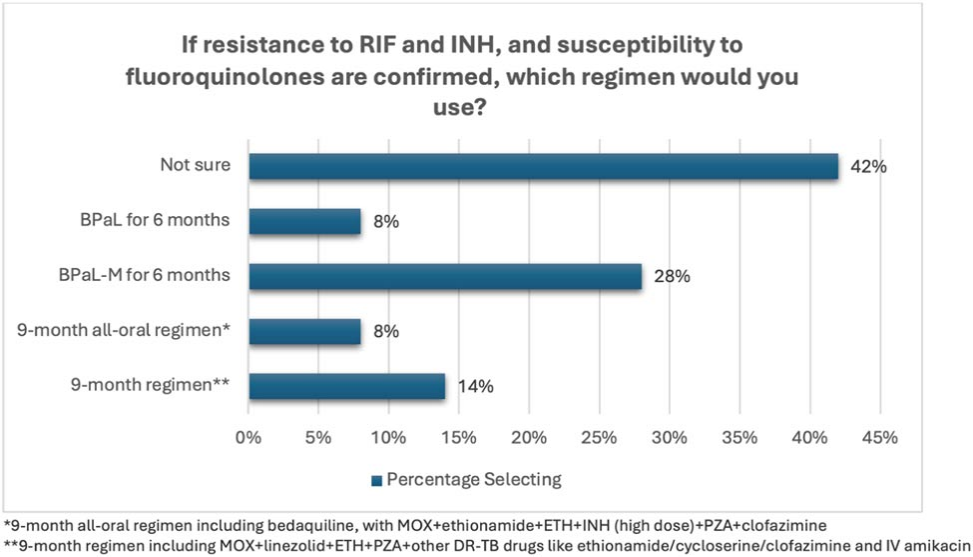
Participant selected approaches to managing drug resistant TB

**Results:**

349 clinicians completed the survey. 93% of respondents preferentially opted for CDC-recommended shorter regimens for TB infection, with only 12% expressing concerns with treatment effectiveness.

In contrast, only 1% selected HPMZ for pulmonary DS-TB and only 5% reported having experience using HPMZ. For confirmed DR-TB, 39% reported they would use either BPaL or BPaL-M, although 40% were unsure about regimen choice. 43% reported uncertainty about effectiveness of shorter regimens for both DS-TB and DR-TB disease.

Qualitative analysis highlighted barriers to the use of shorter regimens for TB disease, including treatment toxicities related to HPMZ or linezolid-based DR-TB regimens, medication interactions such as antitretroviral treatment, and availability of rifapentine and bedaquiline.

**Conclusion:**

While ID physicians are preferentially using shorter regimens for TB infection, uptake of the shorter regimens for TB disease, particularly the 4-month HPMZ regimen is low, due to concerns about effectiveness as well as treatment toxicities. Shared decision making with patients is important to balance the trade-off between the benefits of shorter regimens with enhanced management of potential toxicities.

**Disclosures:**

Philip M. Polgreen, MD, Eli Lily: Advisor/Consultant|Pfizer: Grant/Research Support Maunank Shah, MD, PhD, Scene Health: license of IP

